# Supplementary material for: Phytochrome-Interacting Proteins
Source: Biomolecules. 2023 Dec 21;14(1):9. doi: 10.3390/biom14010009 (PMC10813442; doi:10.3390/biom14010009)
Supplement: Supplementary file 1 [file biomolecules-14-00009-s001.zip › biomolecules-2768941-supplementary.pdf]

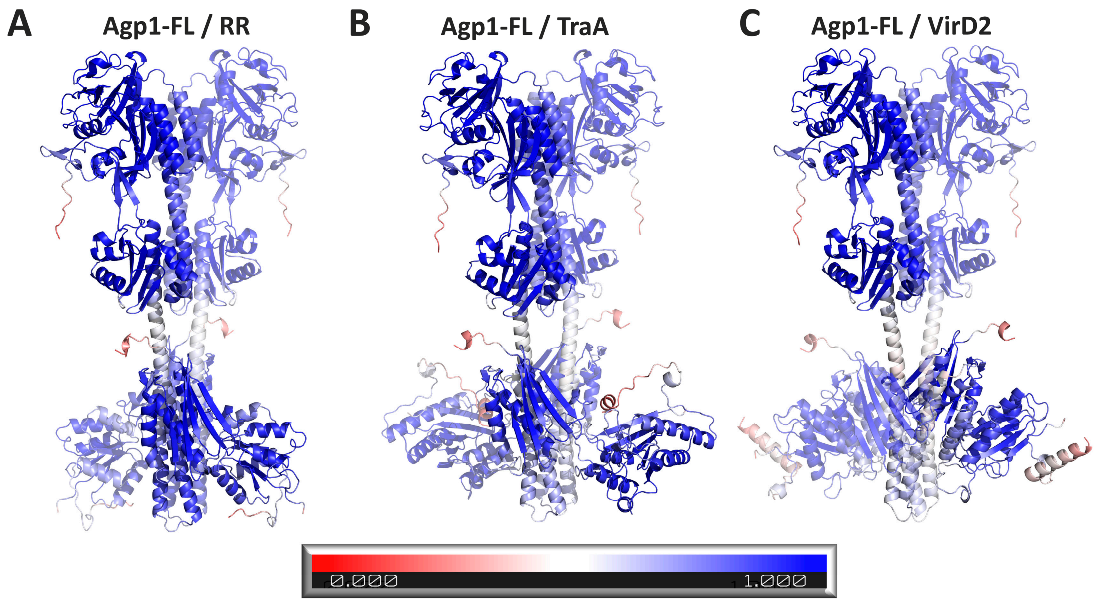

Figure S1. Confidence (pLDDT) values of the AF models presented in Figure 5. The values are scaled from 0 to 1, and the color scale is given below. The models are the same as in Figure 5A–C and in the same order. Almost all regions of the proteins must be regarded as highly confident.
